# A Systematic Literature Review to Compare Clinical Outcomes of Different Surgical Techniques for Second Branchial Cyst Removal

**DOI:** 10.1177/00034894211024049

**Published:** 2021-06-17

**Authors:** Sebastiaan Meijers, Rutger Meijers, Erwin van der Veen, Maaike van den Aardweg, Hanneke Bruijnzeel

**Affiliations:** 1 Department of Otorhinolaryngology and Head and Neck Surgery, University Medical Center Utrecht, the Netherlands; 2 Department of Neurology, Radboud University Medical Center, Nijmegen, The Netherlands; 3 Brain Center Rudolf Magnus, Utrecht, The Netherlands; 4 Central Military Hospital, Utrecht, The Netherlands; 5 Department of Otorhinolaryngology and Head and Neck Surgery, Rivierenland Hospital, Tiel, The Netherlands

**Keywords:** second branchial cyst, congenital anomalies, surgical treatment, endoscopic surgery

## Abstract

**Objective:**

During the last 2 decades, new treatment methods have been developed for the surgical removal of second branchial cysts which result in less visible scars. The aim of this systematic review is to assess which surgical technique for second branchial arch cyst removal results in the lowest complication and recurrence rates with the highest scar satisfaction.

**Methods:**

Two authors systematically reviewed the literature in the Cochrane, PubMed, and EMBASE databases (search date: 1975 to December 2nd, 2020) to identify studies comparing surgical outcomes of second branchial arch cyst removal. Authors appraised selected studies on directness of evidence and risk of bias. Results are reported according to Preferred Reporting Items for Systematic Reviews and Meta-Analyses statement.

**Results:**

Out of the 2442 retrieved articles, 4 articles were included in the current review including a total of 140 operated cysts. Only 2 studies included pre-operatively infected cysts. Follow up ranged from 3 to 24 months. Complication rates ranged from 0 to 27.3% (conventional: [0–10.4%]; endoscopic/retro-auricular: [0–27.3%]). None of the patients presented with postoperative recurrence. Significantly higher scar satisfaction was found in adult patients who underwent endoscopic or retro-auricular hairline incision cyst removal.

**Conclusion:**

No recurrence of disease occurred during (at least) 3 months of follow up using either conventional surgery or endoscopic/retro-auricular techniques. Although more (temporary) complications occur using endoscopic and retro-auricular techniques, patients report a significantly higher scar satisfaction 3 to 6 months after surgery in comparison to the conventional technique. Future studies are needed to support these findings.

## Introduction

The branchial arches consist of clefts and pouches and are the embryological precursors of the face, neck, and the pharynx. In total, 6 pairs of branchial arches form on either side of the pharyngeal foregut. Incomplete obliteration of these arches can lead to formation of branchial arch anomalies, of which second branchial arch anomalies (SBAAs) represent up to 95% of the cases.^1^ The second branchial arch forms part of the hyoid and surrounding structures of the head and neck, while the second branchial pouch shapes the palatine tonsil and the supratonsillar fossa.^2^ Therefore, SBAAs can occur anywhere along the course of the second branchial arch tract that extends from the skin overlying the supraclavicular fossa up to the pharynx at the level of the tonsillar fossa.^1^

Second branchial cysts (SBCs) are the most common SBAAs in adults, whereas sinuses, fistulas and cartilaginous remnants are typically identified in children.^1-3^ Most frequently, cysts present as a asymptomatic neck swelling, however, in around one-third of the cases SBAAs present as a rapid progressive mass due to inflammation.^4,5^ In adults, when encountering an unilateral swelling of the neck, a cystic metastasis of head and neck cancer should always be excluded before SBC diagnosis can be confirmed.^6-8^ Since SBCs are prone to recurrent infections that do not resolve spontaneously, early and complete surgical excision is the recommended treatment.^2,9^ Different surgical techniques for SBC removal have been proposed. Traditionally, conventional surgery using a large cervical incision was used to ensure complete removal.^10-31^ However, the large cervical incision results in a prominent scar. In an attempt to reduce visible scars, newer techniques have been developed, such as endoscopic surgery^9,32-35^ and the use of a retro-auricular hairline incision (RAHI).^35-38^ RAHI can be performed either as an open procedure using a “facelift” incision or as an endoscopic technique. To provide insight in the optimal surgical management of patients presenting with a SBC, this systematic review evaluates which surgical technique (conventional, endoscopic or RAHI) for SBC removal results in the lowest recurrence and complication rates with the highest scar satisfaction.

## Methods

### Search Strategy and Study Selection

**Figure 1.**
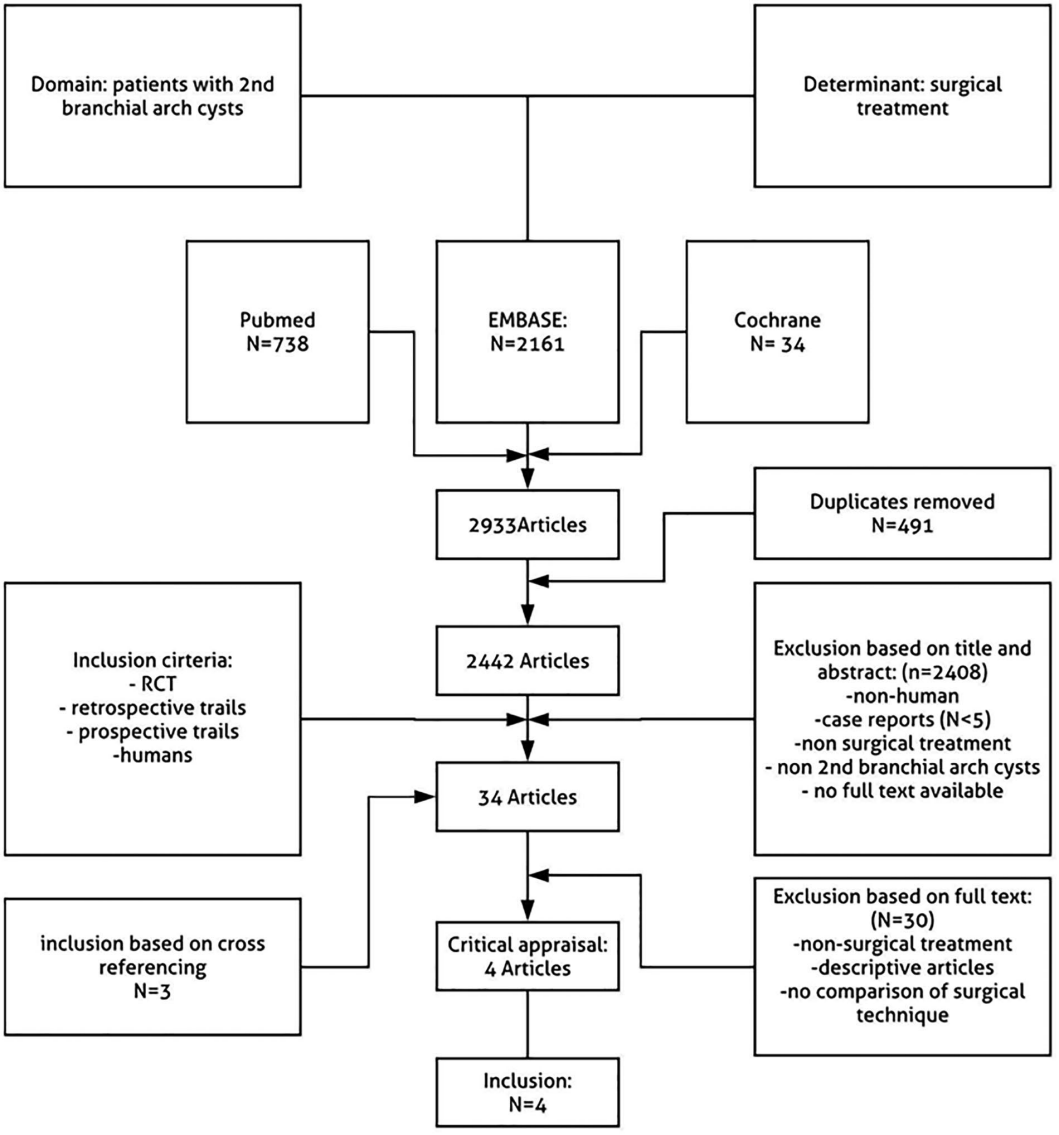
Flow-chart demonstrating the selection of articles from the literature describing surgical second branchial cyst removal.

A systematic literature search was conducted on the 2nd of December 2020, in the PubMed, Cochrane, and EMBASE databases to identify articles comparing outcome data from different surgical techniques for SBC removal (syntax provided in Appendix 1). No restrictions regarding publication data and language were applied. Two authors (S.M., R.M.) independently screened the retrieved articles on title and abstract using pre-defined inclusion and exclusion criteria (Figure 1). The selected articles were read in full-text by the aforementioned 2 authors. The reference lists of the selected articles were reviewed for a cross-reference check to select relevant studies that were not identified in the initial search. All authors were involved in the discussion leading to final article inclusion. Disagreement between authors was resolved by discussion. This study is reported according to the Preferred Reporting Items for Systematic Review and Meta-analysis (PRISMA) statement.^39^

### Critical Appraisal of Topic (CAT)

Four authors (S.M., H.B., E.v.d.V. and M.v.d.A.) critically appraised selected articles regarding directness of evidence (DoE) and risk of bias (RoB) (Table 1). We assessed the DoE using 3 criteria: (1) domain (SBC inclusion) (2) determinant: comparison of 2 or more surgical techniques for cyst removal, and (3) surgical outcome: report on recurrence and complication rates. Overall DoE was rated as high (H), moderate (M), or low (L). Only studies with a high DoE were selected for final inclusion. To perform RoB assessment on the selected studies, authors applied an appraisal tool derived from the Cochrane risk of bias Tool.^40^ Each criterion was rated satisfactory (•), partly satisfactory (○), or unsatisfactory (-) (explanatory legend of Table 1). No studies were excluded based on RoB, adhering to the Grading of Recommendations Assessment, Development, and Evaluation (GRADE) system.^41^

**Table 1.** Critical Appraisal of Topic.

Study	Study design	Directness of evidence					Risk of bias					
		Sample size (n)	Domain	Determinant	Outcome	DoE total	Patient selection	Allocation concealment	Blinding	Incomplete outcome	Follow up	Selective reporting
Chen et al^32^	RCT	25	•	•	•	H	**-**	•	**-**	•	**○**	•
Chen et al^35^	RCT	41	•	•	•	H	**-**	•	**-**	•	**○**	•
Ahn et al^38^	PT	30	•	•	•	H	•	**-**	**-**	•	**-**	•
Iaremenko et al^9^	PT	44	•	•	•	H	**-**	**-**	**-**	•	**○**	•

Abbreviations: NA, not applicable; PT, prospective trial; RCS, retrospective case study; RCT, randomized controlled trail.

Symbols: satisfactory (•), partly satisfactory (○), or unsatisfactory (-).

### Data extraction

The same authors who performed CAT evaluation, extracted relevant data from the included studies (Table 2). The extracted data contained: year of publication, number of included patients (total and patients with SBC specifically), occurrence of bilateral anomalies, pre-operative SBC infection, gender, age at surgery, pre-operative imaging with: computed tomography (CT), magnetic resonance imaging (MRI) or ultrasound (US), operation technique, operating time, incision type and length, follow up duration, recurrence and complication rates, and scar satisfaction. Pooling of data was considered in case of homogeneity between studies (if *I*^2^ was <50%).^42^

**Table 2.** Results of the Included Studies (n = 4) Comparing Conventional Surgery to Endoscopic/RAHI Techniques.

Study	Chen et al^32^		Chen et al^35^		Ahn et al^38^		Iaremenko et al^9^	
Incision type	Cervical incision	Endoscopic RAHI	Cervical incision	Endoscopic lateral neck incision	Cervical incision	Open RAHI	Cervical incision	Endoscopic occipital incision
Patients	12	13	20	21	17	13	22	22
Sex (male/female)	5/7	6/7	9/11	8/13	9/8	2/11	5/17	7/15
Age (years) (median) [range]	31.7(± 2.9)	26.0 (± 11.9)	32 (± 11)	29 (± 8)	34.3 [19-64]	30.5 [17-47]	30.4 ± 11.4	31.6 ± 10.8
Follow up (months)	16(6-24)	16(6-24)	16(6-24)	16(6-24)	3	3	6	6
Scar satisfaction	6.2 ± 0.8*	9.2 ± 0.6*	6.4 ± 0.5*	8.0 ± 0.8*	6.2 (4-8)**	8.8 (7-10)**	79.1 ± 12.0***	97.6 ± 18.5***
Incision length (cm)	NR	NR	6.4 ± 0.5	2.7 ± 0.3	NR	NR	5.1 ± 0.9	5.5 ± 0.6
Operating time (minutes)	49.6 ± 6.9	54.6 ± 6.3	94 ± 21	83 ± 18	68 (45-90)	84 (60-140)	85 ± 15	65 ± 13
Complications								
Recurrence	0%	0%	0%	0%	0%	0%	0%	0%
Seroma/hematoma	0%	0%	0%	0%	11.8%	7.7%	4.5%	0%
Infection	0%	0%	0%	0%	0%	0%	0%	0%
Temporary hypoesthesia of the earlobe	0%	7.7%	0%	0%	0%	23.1%	NR	NR
Temporary pain and difficulty at sideward raising of the arm	NR	NR	NR	NR	NR	NR	4.5%	27.3%

Abbreviations: NR, not reported; RAHI, retro auricular hairline incision.

* Scar satisfaction was measured using a visual analog scale ranging from 0 to 10 6 months after surgery.

** Scar satisfaction was measured using a visual analog scale ranging from 0 to 10 3 months after surgery.

*** Scar satisfaction was measured using the questionnaire “Attitude to health” by R.A. Berezovskaya^43^ 6 months after surgery. The criteria “emotional component” was selected for evaluation of subjective satisfaction with incision scar.

## Results

### Search and selection

**Figure 2.**
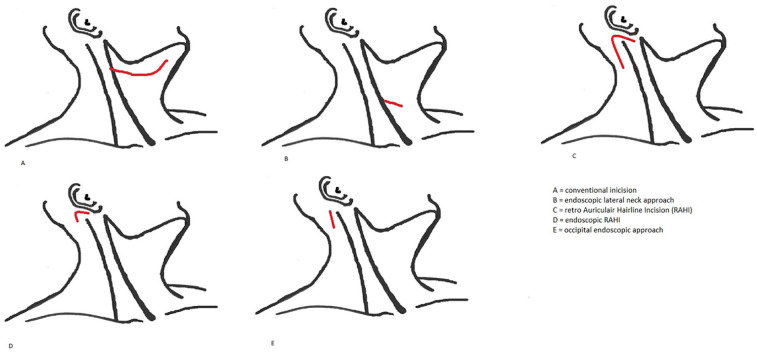
Overview of used surgical incisions.

Following removal of duplicates, we performed title and abstract screening of 2442 articles resulting from our literature search. Thirty-one articles met the predefined inclusion and exclusion criteria and were read full text (Figure 1). Cross-reference of selected articles led to retrieval of 3^15,17,19^ additional eligible articles. Four articles were included for CAT and final inclusion, which resulted in the inclusion of the treatment of 140 cysts. No patients with bilateral cysts were included. These 4 studies^9,32,35,38^ contained 2 randomized controlled trails (RCTs) and 2 prospective trials. The included studies compared the conventional surgical technique to an endoscopic or RAHI technique within the same patient cohort. Figure 2 and Appendix 2 provide an overview of the included surgical techniques. The inclusion dates of the patient cohorts of Chen et al^32^ and Chen et al^35^ did not overlap and therefore, both studies were included in the current review. Pooling of data was not performed in this review due to heterogeneity regarding: baseline characteristics, study design, and applied surgical techniques.

### Data Extraction: Studies Comparing Conventional Surgery to RAHI or Endoscopic Surgery

Table 2 shows the data extraction of 4 included studies that directly compared outcomes between conventional surgery and modern removal techniques in patients presenting with unilateral SBCs. All patients from these studies underwent pre-operative imaging using CT-scan or ultrasound scanning and pre-operative fine needle cytology to confirm the diagnosis (*data not shown*). Chen et al.^35^ compared SBC removal results between conventional, curvilinear, cervical incisions along a natural skin crease (3-4 cm below the lower border of the mandible) to the endoscopic RAHI technique. Adult patients were randomly assigned between both techniques (Table 2). None of the included patients suffered from a pre-operative SBC infection. No recurrence occurred during a follow up of at least 6 months. There was no significant difference in operating time between both techniques; however, there was a significantly (*P* *≤* .001) higher scar satisfaction rate in the RAHI group. This scar satisfaction was measured 6 months postoperatively using a visual analog scale ranging from 0 to 10. Chen et al^32^ compared SBC removal using a curvilinear cervical incision along a natural skin crease (4-5 cm below the lower border of the mandible) to an endoscopic approach of the lateral neck using 2 randomly assigned patient groups. Twenty adult patients were assigned to the conventional cervical incision, whereas 21 patients were assigned to the endoscopic lateral neck approach. Specifics of location and size of the incision were not included in the paper. None of the included patients suffered from a pre-operative SBC infection. No recurrence occurred during a follow up of at least 6 months. Although no significant difference in operating time was reported between both groups, incision length and scar satisfaction did significantly (*P* < .05) differ in favor of the endoscopic technique. This scar satisfaction was (also) measured 6 months postoperatively using a visual analog scale ranging from 0 to 10. Ahn et al^38^ compared SBC removal outcomes between a conventional approach (by making a curvilinear incision directly over the anomaly) and an open RAHI approach in a prospective case control study. Thirteen adult patients were operated by the open RAHI approach while 17 adult patients underwent a (conventional) cervical incision. Ahn et al^38^ reported a pre-operative SBC infection rate of 30.8% in the patients who were operated using the open RAHI technique. No recurrence occurred during a follow up of 3 months. Of the patients who underwent conventional surgery, 11.8% suffered from a postoperative hematoma or seroma, compared to 7.7% of the patients who underwent open RAHI surgery (*non-significant difference*). Only patients of the open RAHI group suffered from postoperative neurological damage that spontaneously resolved (23.1%). The retro-auricular approach entailed significantly longer operating time (*P* = .019), however, resulted in significantly higher scar satisfaction (*P* ≤ .001). Aforementioned scar satisfaction was (also) measured 3 months postoperatively using a visual analog scale ranging from 0 to 10. Iaremenko et al^9^ compared SBC removal outcomes between a conventional approach (by making a skin incision 2.0 to 2.5 cm below the lower border of the mandible) and an endoscopic occipital approach using a controlled study design. The latter technique is comparable to the endoscopic RAHI technique of Chen et al^35^ from a surgical perspective. Twenty-two adult patients were operated by the occipital endoscopic approach, while 22 adult patients underwent a (conventional) cervical incision. No recurrence occurred during a follow up of 6 months. Of the conventional group, 4.5% developed a hematoma and 4.5% developed temporary neurological damage. In the endoscopic occipital approach group, 27.3% reported temporary pain and difficulty at sideward arm raise. Iaremenko et al^9^ reported that aforementioned symptoms in both surgical groups resolved in all cases within 3 months following the surgery. The endoscopic approach resulted in a significantly higher scar satisfaction (*P* *=* .05), but took significantly longer in theatre (*P* *=* .05). Scar satisfaction was measured 6 months postoperatively using the criteria “emotional component” of the “Attitude to health” questionnaire.^43^ Since no recurrence was reported in any of the included studies, no data regarding revision surgery were retrieved.

## Discussion

### Summary of Findings

In this systematic literature review, we compared the clinical outcome (complication and recurrence rates and scar satisfaction) of SBC removal between conventional surgery and less invasive removal techniques (endoscopic surgery or open/endoscopic RAHI). Only 4 studies^9,32,35,38^ were identified that compared the conventional technique with newer techniques within 1 patient cohort. All of these included studies are of low quality due to short follow up, small patient cohorts and a study design prone to bias due to: selection criteria (eg, no inclusion of pre-operatively infected cysts) and lack of blinding. Since evidence is scarce, it remains difficult to provide evidence-based surgical treatment advice.

Results demonstrate that surgical treatment of SBC results in a complication rate ranging from 0 to 27.3% (Table 2). The most reported complications in patients who underwent endoscopic or open RAHI surgery were: temporary earlobe hypoesthesia (7.7-23.1%)^32,38^ (most likely due to perioperative greater auricular nerve manipulation) and temporary pain and difficulty of sideward arm raise (27.3%)^9^ (most likely resulting from spinal accessory nerve manipulation). In patients undergoing the cervical excision technique only 4.5% reported temporary pain and difficulty of sideward arm raise. This relative difference within one studied cohort could indicate that application of newer techniques could result in a greater risk of (temporary) cranial nerve XI injury. Surgical treatment provides a definitive treatment with no reported recurrence using either one of the techniques. Studies that compared both techniques within the same adult patient cohort demonstrated that both the (endoscopic) RAHI approach as well as other endoscopic techniques resulted in high(er) scar satisfaction. Therefore, available evidence demonstrates that application of less invasive SBC removal techniques to treat uninfected second branchial cleft cysts results in relatively higher, temporary complication rates, however, with a significantly higher scar satisfaction. An interesting result, since the operating area is in a prominently visible location in a patient population containing young adults.

Two included studies^32,35^ excluded patients presenting with fistulas and sinuses, pre-operatively infected SBCs and patients who underwent prior neck surgery or radiotherapy. Only Ahn et al^38^ reported on open RAHI treatment of patients with pre-operatively infected SBCs. Although 30.8% of these patients suffered from a pre-operative infection, no relatively higher complication rate was reported for this population compared to the cervical incision group. Iaremenko et al^9^ did not report whether any pre-operatively infected SBCs were included in their study cohort.

### Comparison with Other Studies and Techniques

This is the first systematic literature review reporting on studies assessing the clinical outcome of SBC removal comparing different surgical techniques within 1 cohort. Cohort studies^33,36,37^ investigating only either open/endoscopic RAHI procedures found similar results: absence of recurrence in combination with low complication rates, with an average follow up of (at least) 6, 14.5 and 42 months respectively. The only reported complications in open RAHI surgery were temporary hypoesthesia of the earlobe and hypertrophic scars. Similarly, temporary hypoesthesia^32,38^ of the earlobe was reported (only) in these newer surgical techniques in the comparative studies included in our review (see Table 2).

The conventional second branchial arch anomaly removal techniques have been intensively studied. Table 3 shows an overview of these conventional studies that were identified through the same literature search as we used in the current review. This Table also includes patients (mostly children) presenting with fistulas and sinuses. Table 3 shows that most studies lacked data regarding the: distribution of (included) cysts, sinuses and fistulas, side of the anomalies, description of the used surgical technique or duration of follow up. Only retrospective studies were identified with a complication rate ranging from 0 to 32% and a recurrence rate ranging from 0 to 4.9%. These complication rate percentages are in line with our comparative studies (0-27.3%). However, the recurrence rates are higher, since our selected 4 studies all reported a recurrence rate of 0%. The follow up of the included studies in this review ranged from 3 to 24 months, whereas, the follow-up of these non-comparative studies lasted till 4 or even 10 years.^17,20^ Therefore, the follow up in our selected studies could be too short to identify complete recurrence rates following surgery. Long-term recurrence rates are of major importance because disease recurrence will cause high morbidity and can make revision surgery relatively more complex. Furthermore, this short follow up could also affect the reported scar satisfaction rate, since 3 to 6 months after surgery the final scar result might not be visible yet.

**Table 3.** Non-Comparative Studies Using Conventional Surgery Techniques for Removal of Second Branchial Arch Anomalies (Including Cysts, Fistulas, and Sinuses).

Study	Study design	Used surgical technique	Patients with 2nd arch anomaly (total)	Cyst-fistula-sinus total	Side (L-R-B)/sex (M-F)	Age at surgery (years	Follow-up (months)	Recurrence	Complications
Queizan et al^46^	RCS	Fistula: elliptical incision Cyst: cervical, transversal incision	48 (52)	11-19-13 (17 remnants)	B: 7/27-25	1-7	NR	2%	NR
Doi et al^29^	RCS	“Surgical excision”	44 (58)	7-20-12-39	NR/32-26*	Fistula <5 Cyst >9	NR	2.3%	0%
Takimoto et al^21^	RCS		36 (42)		23-19-0/20-22	5-72			
Ford et al^18^	RCS	68/98 conventional 30/98 stepladder	98 (106)	90-2-?-98**	40%-60%-6/45-53	<13 years	NR	3%	NR
Perez et al^17^	RCS	“Cystectomy”	19 (32)	19-0-0-19♦	NR/11-21	23.9	4 years	6.3%	9.4% wound infection
Atlan et al^23^	RCS	“Local excision”	17 (20)	NA	NR/11-6	2-60 months	NR	NR	11.8% hypertrophic scar
Agaton-Bonilla et al^27^	RCS	Wide, transverse cervicotomy	137 (183)	113-24-0-137	58-123-2/43-98	Cyst mean 23.6 Fistula mean 24.6	24	4.9%	2.9% temporary neurological damage 6.6% infection 11.7% hematoma/seroma
Kadhim et al^4^	RCS	“Surgical removal”	39 (39)	39-0-0-39	23-16-0/16-23	Mean 30.3 (16-52)	6 weeks	0%	0%
Karabulut et al^16^	RCS	Stepladder incision	14 (14)	?-?-13-14	6-R-3/6-8	1.5-16 (5.3)	6 years	0%	0%
Rattan et al^47^	RCS	32/52 surgical excision 20/52 surgical excision and fistulogram 10/52 stepladder	52 (52)	0-52-0-52	12-29-11/38-14	1-13 (4.5)	NR	4%	32% methylene spill
Schroeder et al^24^	RCS	Lateral cervicotomy	51 (67)	14-14-23-51	NA/NA	Cyst: 4.9∟ Sinus: 4.5∟ Fistula: 2.8∟	48	3.9%	1.9% temporary neurological damage 1.5% hematoma/seroma 10.4% infection
Mitroi et al^48^	RCS	Lateral cervicotomy	23 (23)	10-0-13-23	NR/11-12	NR	1-5 years	0%	0%
Papadogeorgakis et al^14^	RCS	Lateral cervicotomy	18(18)	18-0-0-18	11-7/10-8	27.8 (21-62)	1-7 years	0%	11.1% seroma
Bajaj et al^13^	RCS	55/62 elliptical incision 7/62 stepladder	62 (80)	NA	16-34-12/30-32	1-14	6 weeks	1.6%	1.6% seroma
Maddalozzo et al^44^	RCS	Elliptical incision (4 cm)	208 (232)	?-28-?-232	0-25-3/11-17	6-131 months	2 years	0%	0%
Zaifullah et al^25^	RCS	Wide horizontal incision/stepladder	11 (26)	11-2-0-13***	7-3-1/5-7	19.6 (4-44)	NR	0%	25% hypertrofic scar
Erikci and Hosgor^12^	RCS	“Surgical resection”	24 (179)	8-16-0-24	11-10-4/9-16♣	0-14	4-120 months	0%	0%
Kajosaari et al^45^	RCS	“Surgical excision”	68 (68)	0-68-0-68	13-49-6/39-29	0-16	NR	0%	2.9% tonsillectomy re-bleed 1.5% disturbing scar
Prasad et al^31^	RCS	“Surgical excision”	17 (34)	8-9-0-17	NR/9-8	NR	NR	NR	5.9% wound infection 5.9% wound gaping 5.9% neurological deficit
Spinelli et al^20^	RCS	Transverse cervical incision	39 (50)	11-27-1-39	NR/21-29	Cyst 9.5 Fistula 5.1 Sinus 3.7	1-10 years	4%	0%
Kalra et al^30^	RCS	“Surgical excision”	94 (94)	8-48-38-94	24-62-8/70-24	3 months-14 years	NR	2.1%	4.2% wound infection
Pacheco-Ojeda et al^10^	RCS	Mid-neck transverse cervicotomy	43 (51)	43-0-0-0	22-22-1****/16-27	31 (4-60)	84 (3-216)	0%	1.9% hypertrophic scar

*Note.* Adult studies, *pediatric studies.*

Abbreviations: NR, not reported; L, left; R, Right; B, Bilateral; M, male; F, Female.

Symbols: ~ RCS = retrospective cohort study *all patients (also including other than 2nd branchial anomalies) ** 90 patients had cleft sinus or cyst, 6 had cleft cartilage remnant

*** (1 cyst and fistela bilateral)

*** *Medial exit site

∟ Average ♦ in only 19/32 patients the perioperative diagnosis of 2nd branchial cyst was made. Recurrence and complications were calculated for 32 patients

♣ Including one patient with an 4th branchial cyst.

### Quality of Evidence and Potential Biases

Since only 3 articles^15,17,19^ were found following cross-reference, we deemed our performed search strategy complete. The overall quality of the included studies was low (IIb -IV regarding the *Oxford Centre for Evidence-Based Medicine guidelines*): only 2 studies used a RCT to compare the clinical outcome between surgical techniques. In these RCTs, selection bias could not be ruled out due to lack of blinding. The quality of evidence regarding SBAA removal was mostly affected by: small patient cohorts resulting in Type II error (i.e., failing to reject a false null hypothesis), short follow up, unclear inclusion criteria and selective reporting.

## Conclusion

This literature review compares the clinical outcome of SBC removal between conventional surgery and endoscopic surgery or open/endoscopic RAHI. Surgical treatment of uninfected SBCs provides a definitive solution with no reported recurrence using either one of the techniques during relatively short follow up (range: [3-24 months]). Endoscopic or (endoscopic) RAHI surgery results in significantly higher scar satisfaction in comparison with the conventional technique in adults, however, causes more temporary complications (0-27.3%). Since follow up was short, recurrence rates could be underreported and scar satisfaction could be affected by not (yet) judging the final scar result. Scar satisfaction and complication rates were eventually major end points in our study since recurrence rates did not differ greatly in the studies found. Large prospective studies with long-term follow up (>5 years) are currently lacking and will be essential to confirm whether newer techniques (endoscopic surgery or open/endoscopic RAHI) indeed result in higher scar satisfaction and less recurrence on the long-term.

## Supplemental Material

sj-docx-1-aor-10.1177_00034894211024049 – Supplemental material for A Systematic Literature Review to Compare Clinical Outcomes of Different Surgical Techniques for Second Branchial Cyst Removal Click here for additional data file. Supplemental material, sj-docx-1-aor-10.1177_00034894211024049 for A Systematic Literature Review to Compare Clinical Outcomes of Different Surgical Techniques for Second Branchial Cyst Removal by Sebastiaan Meijers, Rutger Meijers, Erwin van der Veen, Maaike van den Aardweg and Hanneke Bruijnzeel in Annals of Otology, Rhinology & Laryngology

sj-docx-2-aor-10.1177_00034894211024049 – Supplemental material for A Systematic Literature Review to Compare Clinical Outcomes of Different Surgical Techniques for Second Branchial Cyst Removal Click here for additional data file. Supplemental material, sj-docx-2-aor-10.1177_00034894211024049 for A Systematic Literature Review to Compare Clinical Outcomes of Different Surgical Techniques for Second Branchial Cyst Removal by Sebastiaan Meijers, Rutger Meijers, Erwin van der Veen, Maaike van den Aardweg and Hanneke Bruijnzeel in Annals of Otology, Rhinology & Laryngology

sj-docx-3-aor-10.1177_00034894211024049 – Supplemental material for A Systematic Literature Review to Compare Clinical Outcomes of Different Surgical Techniques for Second Branchial Cyst Removal Click here for additional data file. Supplemental material, sj-docx-3-aor-10.1177_00034894211024049 for A Systematic Literature Review to Compare Clinical Outcomes of Different Surgical Techniques for Second Branchial Cyst Removal by Sebastiaan Meijers, Rutger Meijers, Erwin van der Veen, Maaike van den Aardweg and Hanneke Bruijnzeel in Annals of Otology, Rhinology & Laryngology
