# Supplementary material for: A Systematic Literature Review to Compare Clinical Outcomes of Different Surgical Techniques for Second Branchial Cyst Removal
Source: Ann Otol Rhinol Laryngol. 2021 Jun 17;131(4):435–44. doi: 10.1177/00034894211024049 (PMC8899809; doi:10.1177/00034894211024049)
Supplement: sj-docx-1-aor-10.1177_00034894211024049 – Supplemental material for A Systematic Literature Review to Compare Clinical Outcomes of Different Surgical Techniques for Second Branchial Cyst Removal [file sj-docx-1-aor-10.1177_00034894211024049.docx]

Appendix 2: overview of surgical techniques.

*Conventional approach:* *(Iaremenko, (10)):* An incision is made in the upper third part of the lateral neck, along a skin crease. Usally 2,0-2.5 below the lower border of the mandibule. Incision is made trough the subcutaneous tissue, the fascia superficialis, platysma muscle. External jugular vein is transected and ligated. Fascia media and fascia profunda are dissected. Cyst fluid aspiration is performed if required. Cyst is removed in total. The wound is closed layer by layer using biodegradable sutures. Drainage tube is placed. The wound is covered with a tight aseptic dressing.

*Retro auricular hairline approach (Chen 2009 (38)):* Retro auricular incision was made through the skin, subcutaneous tissue, and platysma

muscle. The incision was made along the post auricular sulcus and hairline, starting from the lower end of the post auricular sulcus, moving upward to the middle or upper third of the sulcus, and then smoothly angulating downward to 0.5 to 1 cm under the hairline. Careful attention to the overlying sternocleidomastoid muscle prevented injury to the great auricular nerve. The skin flap

was elevated just above the sternocleidomastoid muscle onto the carotid triangle. The cysts were exposed anterior and deep to the sternocleidomastoid muscle at the level of the carotid bifurcation. Dull dissection was used to free the attachments of the cysts. The cysts were then completely removed after aspirating luminal contents. All of the cysts were separated easily from the surrounding

normal tissues and removed completely using this approach without tumor spillage. The retro auricular skin flap was repositioned and sutured.

*Endoscopic lateral neck approach* *(Chen 2014,(36)):* A lateral neck incision was made in the skin, subcutaneous tissue, and platysma muscle. The incision was made along the skin line below the lower bound of the cyst. The working space was created by elevating the skin flap with self-designed custom-made retractors to establish a stable operative space. The wound margin was protected by two applications to avoid injury from the ultrasonic scalpel. Dissection using the ultrasonic scalpel was performed to free the attachments of the cyst. When we separated the cyst, we took care to avoid impairing the common carotid artery, internal jugular vein, vagus nerve, hypoglossal nerve, and accessory nerve. The cyst was completely removed. For very large cysts (longest diameter

of 7 cm) decompression was often performed by fluid aspiration and needle pricking.. The wound was closed by a subcuticular suture with 4-0 Dexon, and a small ventricular drainage tube was inserted.

*Endoscopic RAHI: (Chen 2012(33)):* A retroauricular incision was made through the skin, subcutaneous tissue, and platysma muscle. The incision was made along the postauricular sulcus and hairline, starting from the lower end of the postauricular sulcus, moving upward to the middle or upper third of the sulcus, and then smoothly angulating downward to 0.5 cm above

the hairline. The skin flap was dissected under platysma with the help of the 4-mm-diameter endoscope. During this step, careful attention to the overlying sternocleidomastoid muscle prevented injury to the great auricular nerve and external jugular vein. The working space was then produced by elevating the skin flap just above the sternocleidomastoid muscle onto the carotid triangle. The cyst was exposed anterior and deep to the sternocleidomastoid muscle at the level of the carotid bifurcation after carful dissection of the accessory nerve and posterior belly of digastric muscle. Then, dissection using the ultrasonic scalpel was carried out to free the attachments of the cyst, and the cyst was completely removed. In very large cysts (longest diameter 8 cm), decompression was often performed by fluid aspiration and needle-pricking. Finally, the wound was closed by subcuticular suture with 4-0 Dexon, and a small Hemovac was placed for drainage.

*Occipital endoscopic approach: (Iaremenko (10))*

An incision was performed in the occipital hairline region from occipital bone condylar fossa and going along the hairline 1.2 ± 0.3 cm under it. Layer by layer incision of skin and subcutaneous tissue was done. Fascia dissection was performed by means of unipolar and bipolar laparoscopic tools; a ‘‘tunnel’’ was formed under control of a rigid endoscope; ‘‘tunnel’’ vector was directed along the inferior border of the mandible. Anterior border of sternocleidomastoid muscle was visualized; fascia dissection under internal border of sternocleidomastoid muscle was performed, and the muscle was elevated by means of a retractor. Neurovascular bundle fascia was dissected and exposed the cyst fascia with an anterior-inferior adjoining swollen lymphatic node. In larger cysts, the cyst fascia exposed under the internal border of sternocleidomastoid muscle. In all the cases, aspiration of cyst fluid was performed. The fascia was clamped with forceps and excised by means of ultrasound unipolar and bipolar laparoscopic tools. Tube drainage was inserted into the wound on vacuum. The wound was closed layer by layer with biodegradable synthetic loop sutures. The skin incision was closed with polypropylene loop sutures. The wound was covered with tight aseptic dressing.
