# Supplementary material for: A Systematic Literature Review to Compare Clinical Outcomes of Different Surgical Techniques for Second Branchial Cyst Removal
Source: Ann Otol Rhinol Laryngol. 2021 Jun 17;131(4):435–44. doi: 10.1177/00034894211024049 (PMC8899809; doi:10.1177/00034894211024049)
Supplement: sj-docx-2-aor-10.1177_00034894211024049 – Supplemental material for A Systematic Literature Review to Compare Clinical Outcomes of Different Surgical Techniques for Second Branchial Cyst Removal [file sj-docx-2-aor-10.1177_00034894211024049.docx]

Explanatory Legend of table 1: Critical appraisal of studies reporting on surgical treatment of 2^nd^ branchial arch cysts

Assessed Study Aspect:

**DIRECTNESS OF EVIDENCE:**
Domain: -2^nd^ branchial cyst only or 2^nd^ branchial cysts reported separately ●
 - also other branchial arch anomalies included -

Determinant: - comparison of surgical techniques ●
 - unclear description of surgical technique ○
 - no description of surgical technique -

Outcome: - recurrence rate clearly mentioned ●
 - no recurrence rate mentioned -

**RISK OF BIAS:**
1.Patient selection: - Clear inclusion and exclusion criteria ●
 - Unclear inclusion and exclusion criteria -

2. Allocation concealment: - type of treatment is randomly assigned ●
 - treatment is not randomly assigned -
 - Not applicable NA

3. Blinding: - the two treatment groups were blinded for the researchers ●
 - the two treatment groups were not blinded for the researchers -
 - Not applicable NA

4. Incomplete outcome: - <10% loss to follow up ●
 - >10% loss to follow up or unclear -

5. Follow up: - > 1 year ●
- > 6 months ○
- unclear -

6. Selective reporting: - clear definition and description of complications ●
- unclear or no definition and description of complications -

DIRECTNESS OF EVIDENCE: ● = 1 point ○ = 0,5 point - = 0 points
RISK OF BIAS: - = 1 point ○ = 0,5 point ● = 0 points
