# Supplementary figures and images for: A Systematic Literature Review to Compare Clinical Outcomes of Different Surgical Techniques for Second Branchial Cyst Removal
Source: Ann Otol Rhinol Laryngol. 2021 Jun 17;131(4):435–44. doi: 10.1177/00034894211024049 (PMC8899809; doi:10.1177/00034894211024049)

Figure 2: overview of used surgical incisions.


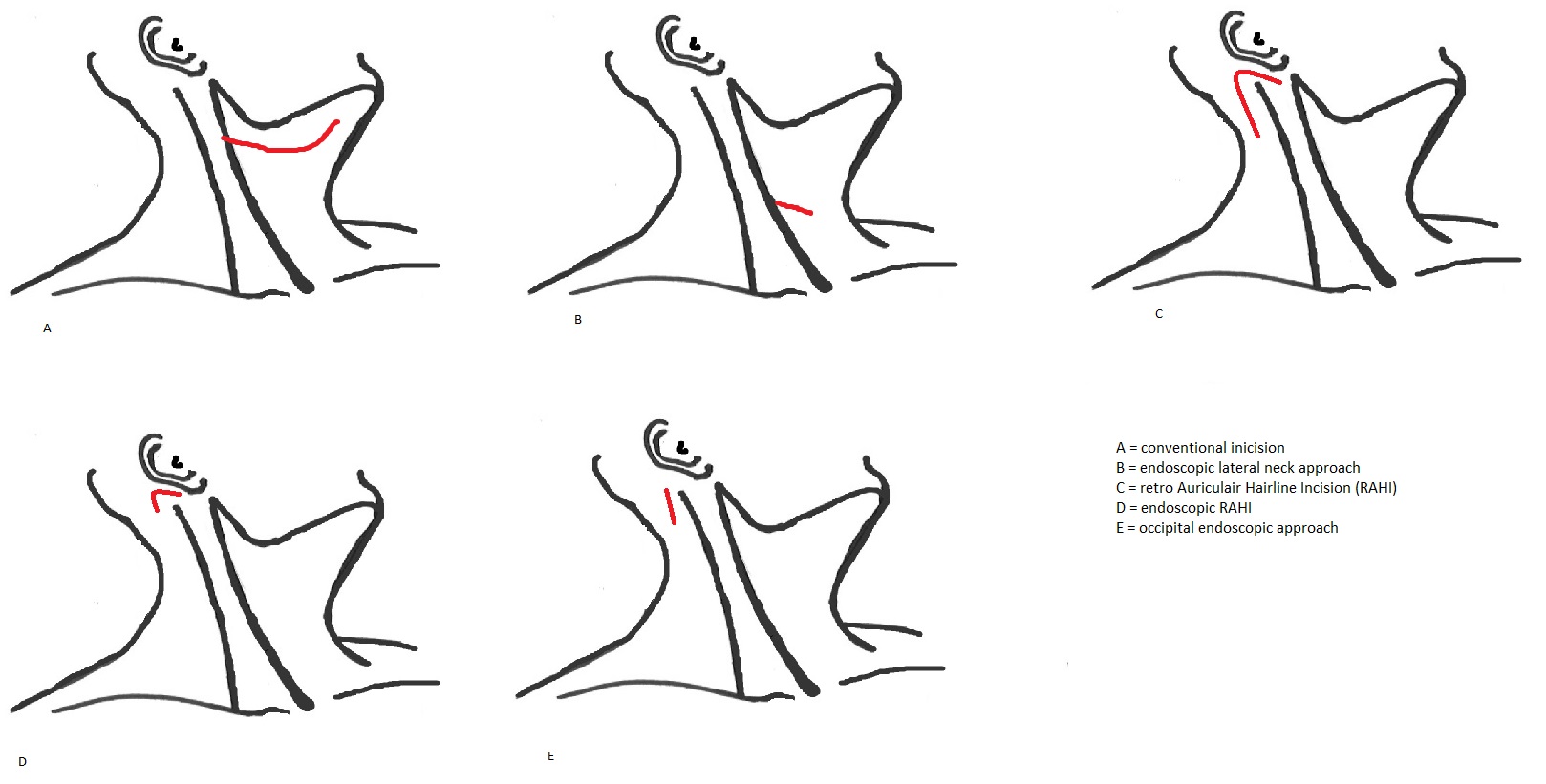

Supplement: sj-docx-3-aor-10.1177_00034894211024049 – Supplemental material for A Systematic Literature Review to Compare Clinical Outcomes of Different Surgical Techniques for Second Branchial Cyst Removal [file sj-docx-3-aor-10.1177_00034894211024049.docx]
